# 1,3-propanediol production with *Citrobacter werkmanii* DSM17579: effect of a *dhaD* knock-out

**DOI:** 10.1186/1475-2859-13-70

**Published:** 2014-05-17

**Authors:** Veerle ET Maervoet, Sofie L De Maeseneire, Fatma G Avci, Joeri Beauprez, Wim K Soetaert, Marjan De Mey

**Affiliations:** 1 Centre of Expertise - Industrial Biotechnology and Biocatalysis, Department of Biochemical and Microbial Technology, Ghent University, Coupure links 653, B-9000 Ghent, Belgium; 2 Present address: Laboratory of Biochemistry and Brewing, Department of Applied Bioscience Engineering, Ghent University, Valentin Vaerwyckweg 1, 9000 Ghent, Belgium; 3 Present address: Bioengineering Department, Faculty of Engineering, Ege University, 35100 Bornova Izmir, Turkey

**Keywords:** *Citrobacter werkmanii* DSM17579 ∆*dhaD*, Glycerol dehydrogenase, 3-hydroxypropionaldehyde, 1,3-propanediol, Yield

## Abstract

**Background:**

1,3-propanediol (PDO) is a substantially industrial metabolite used in the polymer industry. Although several natural PDO production hosts exist, e.g. *Klebsiella* sp., *Citrobacter* sp. and *Clostridium* sp., the PDO yield on glycerol is insufficient for an economically viable bio-process. Enhancing this yield via strain improvement can be achieved by disconnecting the production and growth pathways. In the case of PDO formation, this approach results in a microorganism metabolizing glycerol strictly for PDO production, while catabolizing a co-substrate for growth and maintenance. We applied this strategy to improve the PDO production with *Citrobacter werkmanii* DSM17579*.*

**Results:**

Genetic tools were developed and used to create *Citrobacter werkmanii* DSM17579 ∆*dhaD* in which *dhaD,* encoding for glycerol dehydrogenase, was deleted. Since this strain was unable to grow on glycerol anaerobically, both pathways were disconnected. The knock-out strain was perturbed with 13 different co-substrates for growth and maintenance. Glucose was the most promising, although a competition between NADH-consuming enzymes and 1,3-propanediol dehydrogenase emerged.

**Conclusion:**

Due to the deletion of *dhaD* in *Citrobacter werkmanii* DSM17579, the PDO production and growth pathway were split. As a consequence, the PDO yield on glycerol was improved 1,5 times, strengthening the idea that *Citrobacter werkmanii* DSM17579 could become an industrially interesting host for PDO production.

## Background

Fermentation development and strain improvement are the two most common ways to improve the yield of a biotechnological production process. The first was already described in 1969 when water soluble polymers, such as carboxymethylcellulose and thickening agents, were added to the fermentation medium to enhance the yield [1]. Nowadays, state of the art methods for fermentation development are still used for improving production yields. For example, complex statistical methods are used to optimize and standardize media and fermentation conditions in a more directed way [2,3]. Although classical strain improvement techniques such as mutagenesis, have long time been used, the more targeted approach of metabolic engineering is currently preferred [4]. While for metabolic engineering mainly two strategies were earlier applied, i.e. over expression of genes directly involved in the synthesis of the desired product, and inactivation of competing pathways [5-8], today the toolset to do so has vastly expanded with the emergence of synthetic biology and protein engineering [9].

Strain improvement via metabolic engineering can be used to disconnect the production and growth pathways [10]. In this approach, the resulting strain needs two substrates of which one is exclusively used for product formation, while the second is only consumed for growth and maintenance. Dodge and Valle [10] have uncoupled the productive and catabolic pathways via the inhibition of glucokinase (E.C. 2.7.1.2). Hereby, glucose is used for the production of several metabolites, *e.g*. ascorbic acid, riboflavin, and D-ribose, while fructose or another non-glucose carbon source is used for growth and cell maintenance.

In the case of 1,3-propanediol (PDO) production, uncoupling is achieved when glycerol is entirely used for PDO synthesis and not catabolized for growth and maintenance. PDO has been chosen as target molecule as this is a starting compound for several industrial applications, e.g. composites, adhesives, laminates, mouldings and antifreeze [11]. Moreover, this bifunctional compound is of particular importance in the polymer industry for the synthesis of polyesters (such as polytrimethylene terephthalate), polyethers and polyurethanes [8]. Maervoet *et al*. [12] describe that *Citrobacter werkmanii* DSM17579 wild-type cells use glycerol for growth when co-substrates are added, so it is not far-fetched to assume that uncoupling the pathways could result in higher PDO yields for this new PDO-production host. Furthermore, renewable, cheap co-substrates could be used, *i.e.* lignocellulosic and hemicellulosic hydrolysates or whey, which might result in a reduced final production cost.

Detachment of the PDO production pathway and the growth pathway from glycerol in *Citrobacter* requires (at least) the deletion of the first gene of the oxidative pathway, *dhaD,* encoding for glycerol dehydrogenase (GDH, E.C. 1.1.1.6). In this article, the creation of the strain *C. werkmanii* DSM17579 ∆*dhaD* is described. It was confirmed that this strain is unable to grow on medium with glycerol as the only carbon source under anaerobic conditions. Next, 12 sugars and dihydroxyacetone (DHA) were tested as substrate for growth and their effect on PDO production was examined. Finally, for the most promising co-substrate, several molar ratios co-substrate/glycerol were tested and evaluated for PDO yield on glycerol, PDO titer, and productivity.

## Results and discussion

### Creation of *Citrobacter werkmanii* DSM17579 ∆*dhaD*

The technique used to create *Citrobacter werkmanii* DSM17579 ∆*dhaD* is based on the one-step deletion method described by Datsenko and Wanner [13]. Four major changes have been made to optimize this protocol for *C. werkmanii* DSM17579. Firstly, the helper plasmids to create the knock-out were changed. The strain is resistant to ampicillin up to concentrations of 0,2 g/L, while 0,05 g/L gentamicin completely inhibits growth (Additional file 1: Table S1). Therefore, the plasmids pKD46 and pCP20 were replaced by pKD46-Gm and pCP20-Gm. These last plasmids were kindly provided by Benoît Doublet (INRA, France) [14] and contain the gentamicin resistance gene *aac(3)-Id* of *Salmonella enterica* as pressure. Then, the length of the homology overhangs was doubled to ±50 nt, because it has been proven that longer homology arms improve the transformation efficiency with linear dsDNA [15]. Thirdly, the concentration of L-arabinose was raised to 20 μM, as enhancing the extracellular concentration of L-arabinose, would increase its intracellular concentration and would possibly also improve the expression of *bet*, *exo* and *gam*. Finally, the incubation time after electroporation with linear dsDNA was prolonged to 3 h. During this time, the membrane pores are resealed and the different genes, such as the chloramphenicol and kanamycine resistance markers are expressed. To the best of our knowledge, this is the first time a transformation and knock-out protocol is described for *C. werkmanii.*

### Characterization of *Citrobacter werkmanii* DSM17579 ∆*dhaD*

#### Glycerol as sole carbon source

In first instance, *Citrobacter werkmanii* DSM17579 ∆*dhaD* was grown on glycerol as sole carbon source under anaerobic conditions to verify the knock-out. Up to 53 h after inoculation, no notable growth was detected for the knock-out strain, as expected (start OD of 0.09 ± 0.03, final OD of 0.11 ± 0.02). Due to the deletion of *dhaD*, coding for GDH, the oxidative pathway of the glycerol metabolism is blocked, i.e. the pathway for growth and cell maintenance from glycerol is disabled. As a consequence, the organism only grows in the presence of another C-source. Therefore, in a following step, 12 sugars and DHA were investigated as possible co-substrates for the production of PDO.

#### Sugars as only carbon source

In first instance, growth of the ∆*dhaD* strain in cultivation medium with one of the 12 sugars or DHA as sole carbon source was compared to growth of the wild-type on these substrates. In this way, a possible effect of the deletion of the *dhaD* gene on the sugar metabolism of *C. werkmanii* DSM17579 could be investigated.

**Figure 1:**
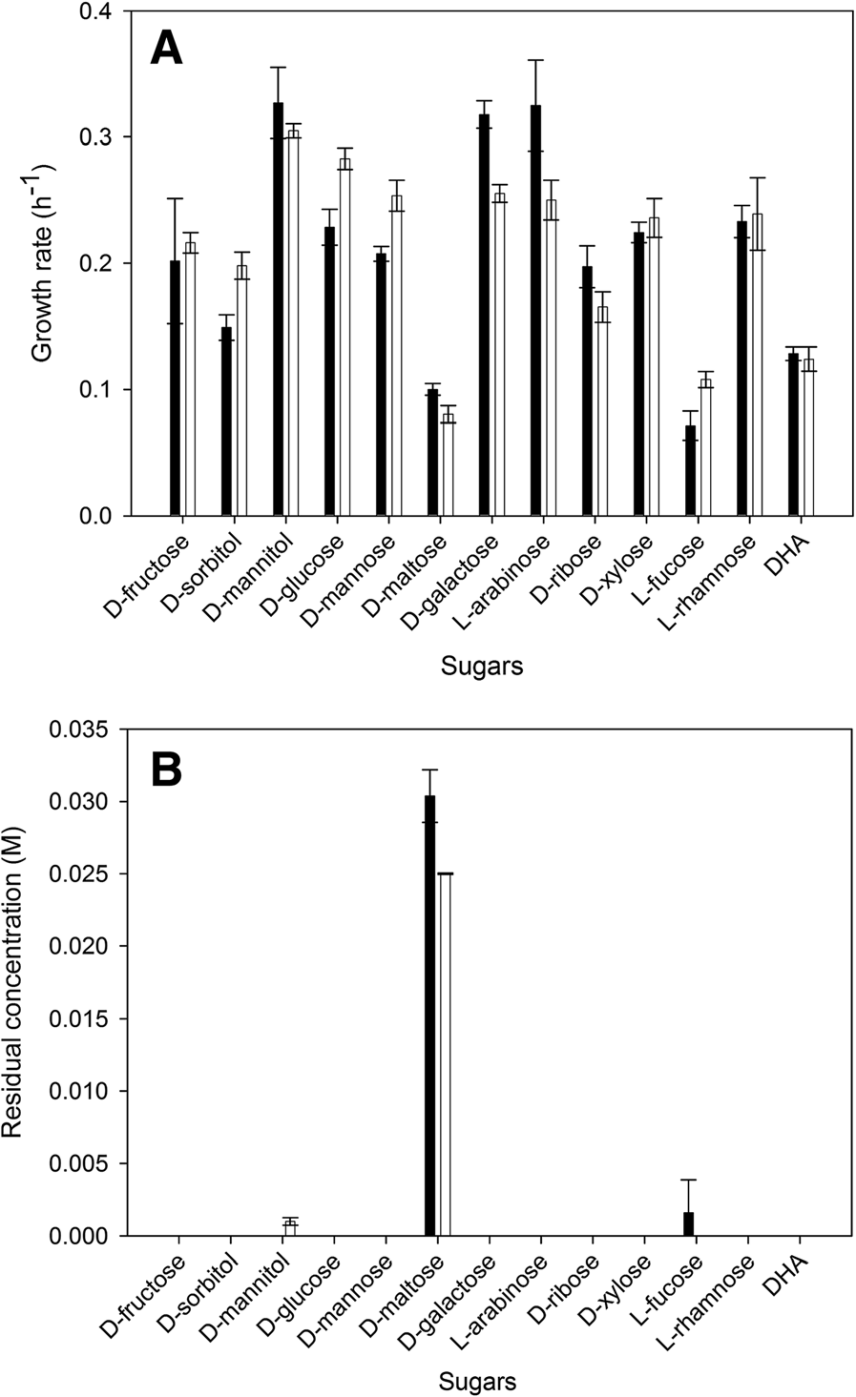
**Difference in growth rate (A) and residual sugar concentration (B) with sugars as only C-source.***C. werkmanii* DSM17579 ∆*dhaD* (black) and *C. werkmanii* DSM17579 (white) were compared for the individual sugars (0.065 M) as the sole carbon source under anaerobic conditions in flask cultures. The errors represent the standard deviation calculated from 2 independent experiments.

Similar as for the wild-type, the mutant can use all 12 sugars and DHA as sole carbon source (Figure 1B). However, an effect on the growth rate is observed (Figure 1A). For D-glucose, D-mannose, L-fucose, and D-sorbitol, the mutant exhibits a lower growth rate as compared to the wild-type. In contrast, for L-arabinose, D-ribose, D-galactose, and D-maltose, the gene deletion has a positive effect on the growth rate. These results indicate that the cheap hemicellulosic hydrolysates, which consist of L-arabinose, D-ribose, D-xylose, D-arabinose, D-galactose, D-mannose and D-glucose [16] may even be a better substrate for the *dhaD* knock-out mutant than for the wild-type strain. For the remaining sugars, no effect of the *dhaD* deletion on the growth rate was detected.

**Figure 2:**
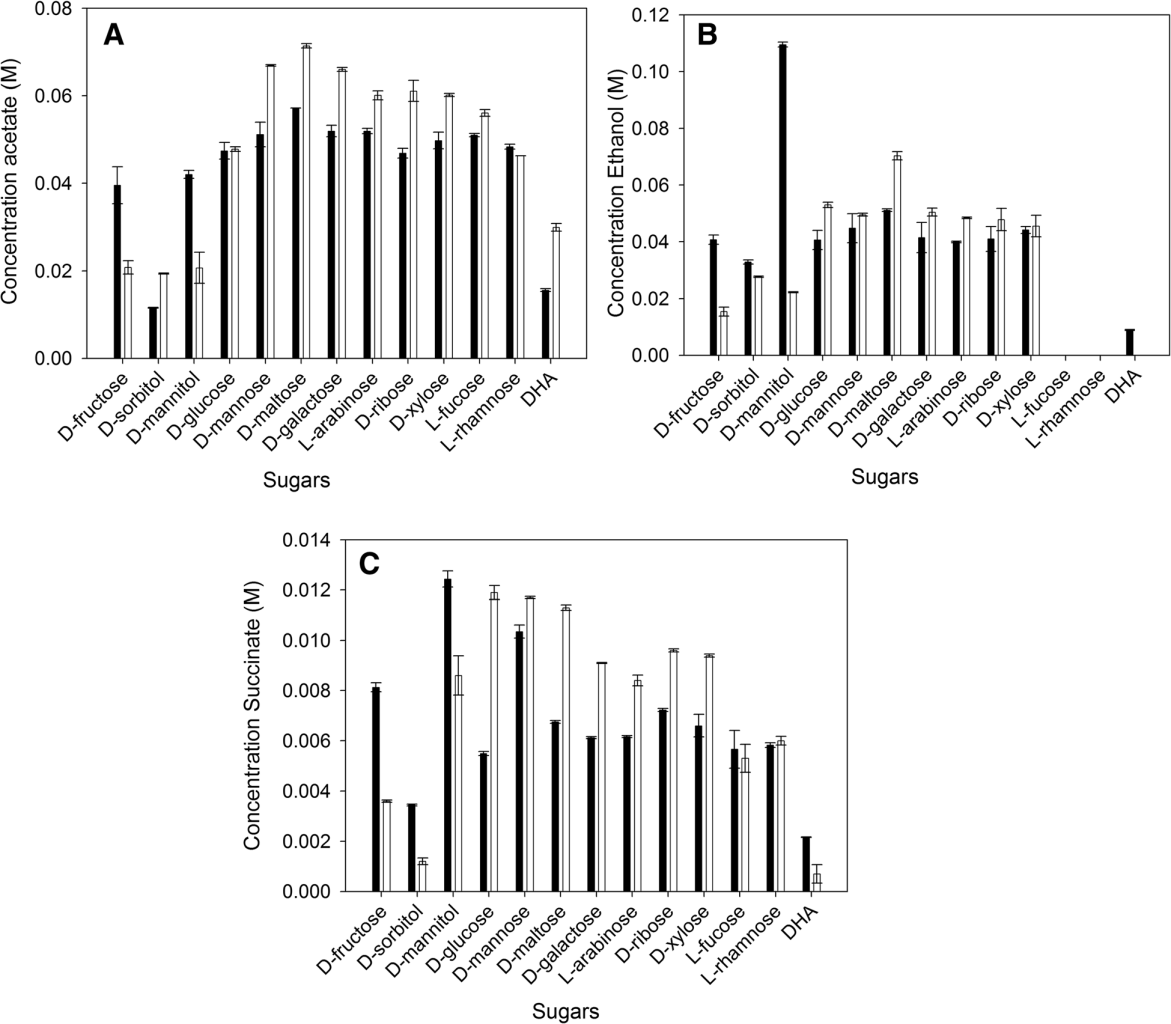
**Comparison of the final concentration acetate (A), ethanol (B), and succinate (C).***C. werkmanii* DSM17579 ∆*dhaD* (black) and *C. werkmanii* DSM17579 (white) were grown in flask cultures under anaerobic conditions in minimal medium with different substrates (0.065 M) as the sole carbon source. The errors represent the standard deviation calculated from 2 independent experiments.

The wild-type *C. werkmanii* DSM17579 does not produce ethanol when it is grown on L-rhamnose, L-fucose or DHA as only carbon source [12,17]. Instead, 1,2-propanediol is produced using L-rhamnose or L-fucose, while 1,3-propanediol is formed starting from DHA. Similar results are obtained when the ∆*dhaD* strain is grown on L-fucose or L-rhamnose: 19.43 ± 0.18 mM 1,2-propanediol is produced on L-fucose and 20.44 ± 0.21 mM on L-rhamnose, while no ethanol is detected (Figure 2). However, in contrast to the wild-type, when the mutant strain is grown on DHA, ethanol is formed and not 1,3-propanediol. This is probably due to the deletion of *dhaD*. When the wild-type is grown on glycerol, glycerol is converted to DHA by GDH. This enzyme may be reversible, so when the same strain is grown on DHA, glycerol is formed which is then further converted via 3-hydroxypropionaldehyde (3-HPA) to PDO by the glycerol dehydratase (GDHt, E.C. 4.2.1.30) and the 1,3-propanediol dehydrogenase (PDODH, E.C. 1.1.1.202), respectively. However, in the mutant strain GDH is not active anymore and thus DHA cannot be converted into glycerol and further into PDO. Hereby, reducing equivalents (NADH), which are normally used by PDODH, become available and can be used to form ethanol.

Just as in the wild type, PDO is not detected when *C. werkmanii* DSM17579 ∆*dhaD* is grown on the other carbon sources. These other sugars can be divided in three clusters according to their metabolic profile (Figure 2). The first cluster consists of D-fructose, D-mannitol and D-sorbitol. They show an increased production of ethanol and succinate, and a variable production of acetate compared to the wild-type strain. This enhanced production of ethanol and succinate may be due to the increased availability of NADH which is a result of the deletion of *dhaD*, as the enzymes to produce ethanol and succinate are NADH-dependent enzymes. D-glucose and D-mannose belong to the second cluster which shows a reduced acetate, ethanol and succinate production due to a reduced growth rate (Figure 1A). This decreased growth rate namely leads to a lessened overflow metabolism resulting in a reduced byproduct rate and thus in a lower byproduct formation [18]. The last cluster consists of L-arabinose, D-ribose, and D-galactose. These non-PTS (phosphoenol pyruvate:carbohydrate phosphotransferase system, T.C. 4.A.1) sugars are more inherent leading to a reduced concentration of byproducts. Hereby, less inhibitory compounds will be formed and thus an increased growth rate can be noticed.

#### Sugars as co-substrates

In order to find a suitable co-substrate for the production of PDO from glycerol with the *dhaD* knock-out, the *C. werkmanii* DSM17579 wild-type and the *dhaD* knock-out mutant were grown anaerobically on glycerol and a co-substrate in a molar ratio co-substrate/glycerol of 0.33. This ratio was chosen, because Xiu *et al.*[19] found that *K. pneumoniae* converts glycerol completely to PDO at a ratio of 0.32 mol glucose/mol glycerol under anaerobic conditions under the condition that glycerol cannot enter the oxidative pathway.

**Figure 3:**
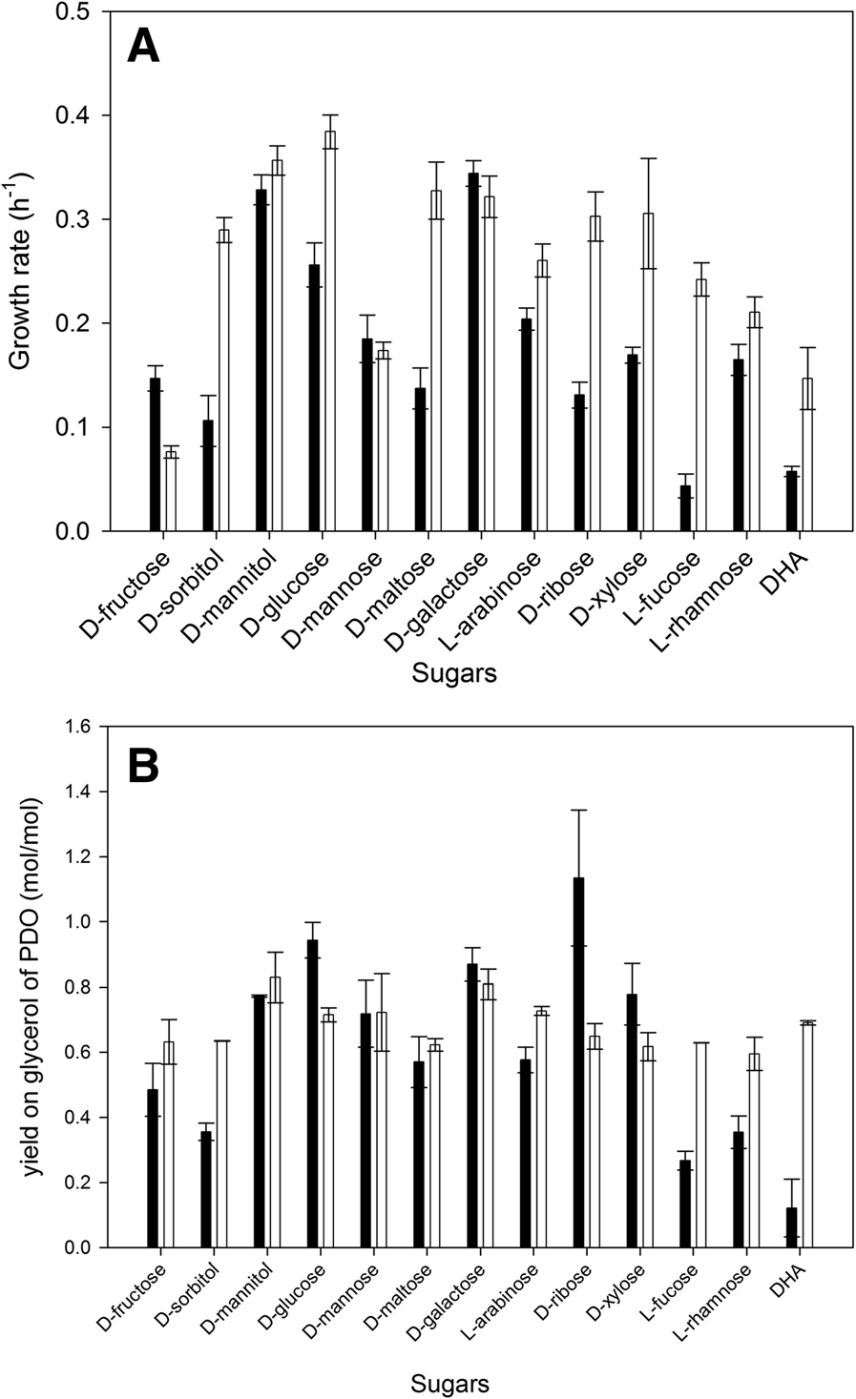
**Comparison of the growth rate (A) and yield on glycerol of PDO (B) using co-substrates.***C. werkmanii* DSM17579 ∆*dhaD* (black) and *C. werkmanii* DSM17579 (white) were grown in minimal medium with glycerol (0.163 M) and different co-substrates (0.33 molar ratio co-substrate/glycerol) on flask scale under anaerobic conditions. The errors represent the standard deviation calculated from 2 independent experiments.

Compared to the wild-type, lower growth rates are observed for almost all sugars (Figure 3A). This may be explained as the wild-type also uses glycerol for growth and cell maintenance, next to the co-substrate, and the growth rate on glycerol is higher than on the tested sugars [12]. For example, when the growth rate of the wild-type is compared between growth on glucose solely or on glucose and glycerol, an increase can be observed from 0.28 ± 0.01 h^−1^ to 0.38 ± 0.02 h^−1^. However, the growth rate does not change when the single knock-out strain is grown on glucose alone compared to growth on glucose and glycerol (respectively, 0.23 ± 0.01 h^−1^ and 0.26 ± 0.02 h^−1^).

**Figure 4:**
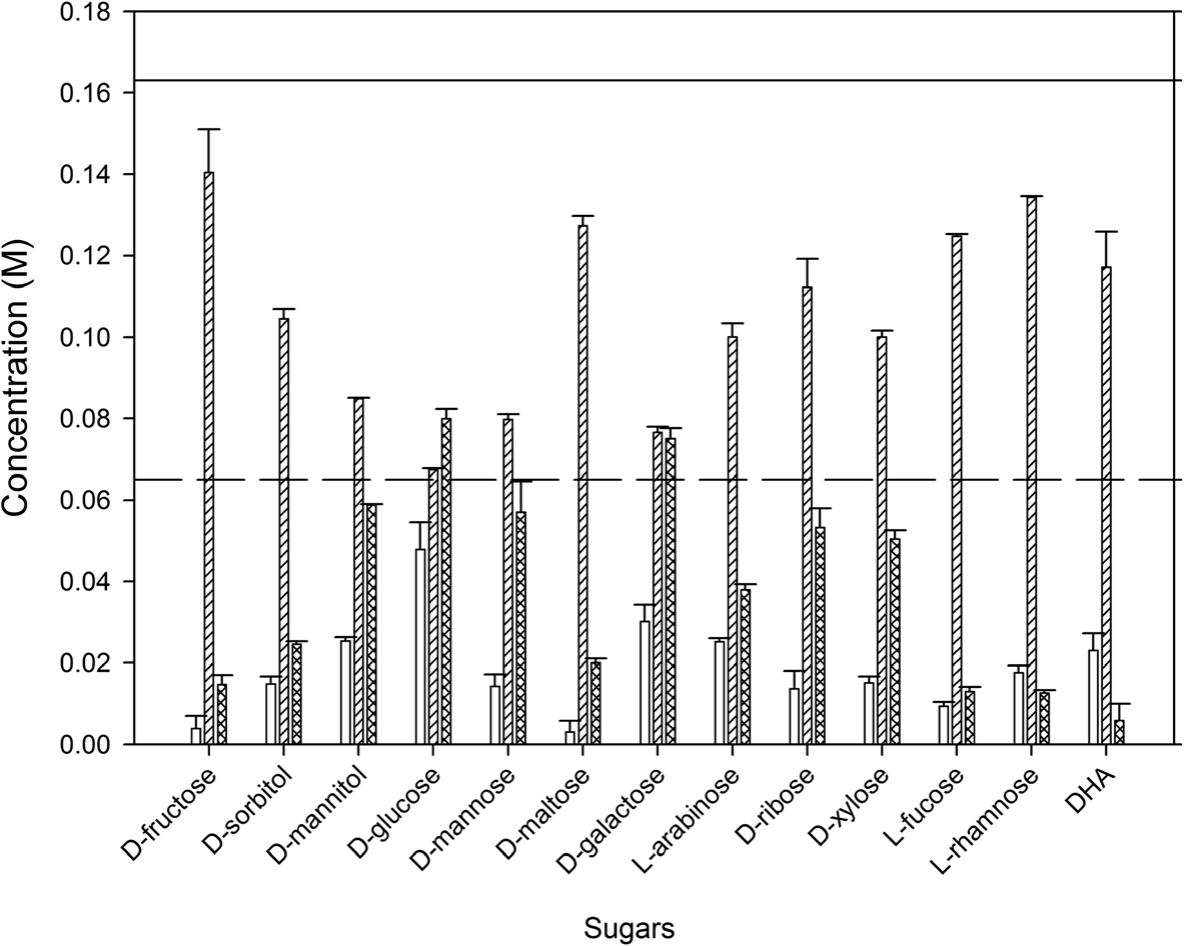
**Consumed co-substrate concentration (blank), residual glycerol concentration (slanted lines) and final PDO concentration (crossed lines).***C. werkmanii* DSM17579 ∆*dhaD* cultures were grown on flask scale under anaerobic conditions in minimal medium with glycerol and different co-substrates (0.33 molar ratio co-substrate/glycerol). The solid line represents the initial glycerol concentration (0.163 M), the dashed line the initial co-substrate concentration (0.065 M). The errors represent the standard deviation calculated from 2 independent experiments.

In none of the glycerol/co-substrate combinations the glycerol and co-substrate is completely consumed (Figure 4). As an excess of the macronutrients is present in the medium, this early utilization stop is not due to N- or P-limitation. Another reason may be a pH drop caused by the formation of ethanol, acetate, succinate and lactate. An increased production of 3-HPA may also be a cause just as noticed for glucose as co-substrate (see further). More than 35% of the co-substrate is consumed in the case of D-glucose, DHA, D-mannitol, D-galactose, and L-arabinose. With these co-substrates, most glycerol is consumed during the growth of the knock-out mutant on D-glucose.

The cultures were further analyzed towards their PDO and by-product formation. The highest final PDO concentration is noticed when D-glucose or D-galactose are used as co-substrate, namely 79.93 ± 2.38 mM and 74.99 ± 2.64 mM, respectively. Although these final titers are reduced compared to the wild-type strain on these co-substrates [12], the yield is increased to, respectively, 0.94 ± 0.05 mol PDO/mol glycerol and 0.87 ± 0.05 mol PDO/mol glycerol, compared to, respectively 0.72 ± 0.02 mol PDO/mol glycerol and 0.81 ± 0.05 mol PDO/mol glycerol for the wild-type. Thus, the reduced final PDO concentration is due to the incomplete consumption of glycerol. Compared to the wild-type strain [12], the yield also increases using D-xylose and D-ribose as co-substrates, indicating that the cutoff of the catabolic pathway has a positive effect on the conversion of glycerol to PDO for these sugars (Figure 3B). It is worthwhile noticing that the maximum theoretical yield (1.0 mol PDO/mol glycerol) has not been reached in any of the co-substrates indicating that the limiting step of the PDO-production pathway is presumably the PDODH. Since this enzyme uses NADH, it may be that not enough NADH is produced or that the activity of other NADH-consuming enzymes is higher (such as ethanol dehydrogenase or lactate dehydrogenase). One solution could be to reduce the activity of the latter enzymes or to even delete the corresponding genes.

The combination of glycerol and a co-substrate results in a decreased production of ethanol, lactate and succinate for most sugars as compared to the co-substrates as sole C-sources (Additional file 1: Figure S1), meaning that there is a competition for the reducing equivalents (NADH) between the production of these compounds and the conversion of 3-HPA to PDO. Therefore, deletion of the genes responsible for the production of ethanol, lactate and succinate could further increase the yield on glycerol of PDO and the final PDO titer.

Da Cunha and Foster [20] have noticed a similar decrease in the production of ethanol and lactate growing *Lactobacillus* species on D-glucose, D-ribose, and D-fructose with or without glycerol. Moreover, they noticed that co-fermentation of glycerol and glucose results in an increase of the final acetate concentration compared to glucose as sole carbon source. The same effect related to acetate was observed when *C. werkmanii* DSM17579 ∆*dhaD* was grown in medium with glycerol and glucose. The other sugars had a negative or no effect on the acetate concentration (Additional file 1: Figure S1).

Finally, the best co-substrate was chosen, considering its price, its metabolic profile, its catabolic repression on glycerol, its production rate and the yield of PDO on glycerol. D-Glucose and D-galactose resulted in an analogous metabolite formation (Additional file 1: Figure S1), and yield. Moreover, their production rates are alike (3.33 ± 0.10 mM/h for D-glucose, 3.13 ± 0.11 mM/h for D-galactose). However, D-glucose is much cheaper than D-galactose: in February 2014, the price for D-glucose was 0.25 €/kg [21], while that of D-lactose (the precursor of D-galactose) was 1,02 €/kg [22]. D-galactose could also be provided as its precursor D-lactose, via whey, a waste stream of the diary industry. However, *C. werkmanii* DSM17579 is unable to grow on D-lactose [12], so the use of whey as co-substrate requires additional modifications in *C. werkmanii* DSM17579 ∆*dhaD,* which goes beyond the scope of this research (*i.e*. overexpression of a β-galactosidase gene to ensure the conversion of D-lactose to D-galactose). Therefore, D-glucose was chosen as the preferred co-substrate for the production of PDO.

### Effect of the dhaD knock-out on the metabolism of C. *werkmanii* DSM17579

To investigate the effect of the *dhaD* knock-out on the metabolism of *C. werkmanii* DSM17579, the wild-type and knock-out strain were grown in shake flasks under aerobic and anaerobic conditions in medium with glycerol as sole carbon source, and with glycerol and glucose in a molar ratio 3 to 1. As mentioned before, *C. werkmanii* DSM17579 *∆dhaD* is unable to grow under anaerobic conditions. However, under aerobic conditions, the single knock-out is still able to grow on glycerol (data not shown). Similar results have been obtained by Horng *et al.*[23] and Seo *et al.*[24] for *Klebsiella* sp. *K. pneumoniae* ∆*dhaD* grows on minimal medium with glycerol as sole carbon source under micro-aerobic conditions due to the activity of glycerol kinase (E.C. 2.7.1.30), which converts glycerol in glycerol-3-phosphate using ATP as cofactor. Glycerol-3-phosphate can then enter the glycolysis, meaning glycerol can be converted via two oxidative pathways in *K. pneumoniae*. Analogously, glycerol kinase is active in *K. aerogenes* NCIB418, in a chemostat culture, under aerobic, carbon-limited conditions, while under aerobic, sulfate- and ammonia-limited conditions and under anaerobic conditions, GDH is the prominent enzyme [25]. As the glycerol kinase gene sequence of *C. werkmanii* DSM17579 is not known yet, A BLAST search (algorithm: blastp; database: non-redundant protein sequences) of the amino acid sequence of the glycerol kinase of *K. pneumoniae* to the known genomes of *Citrobacter* species has been performed. This revealed a hypothetical protein with predicted glycerol kinase activity (Table 1). So, we assume that under aerobic conditions, glycerol kinase is active in *C. werkmanii* DSM17579 ∆*dhaD,* resulting in growth of this strain on minimal medium with glycerol as sole carbon source.

**Table 1:** Percentage identity, E-value and bitscore

**Name**	**% identity**	**E-value**	**Bitscore**
*Citrobacter youngae* ATCC29220	95	0.0	2588
*Citrobacter koseri* ATCC BAA-895	95	0.0	2586
*Citrobacter rodentium* ICC168	95	0.0	2583
*Citrobacter* sp. 30_2	94	0.0	2578

The amino acid sequence of the glycerol kinase of *K. pneumoniae* and the known genomes of *Citrobacter* species were compared.

To study the glycerol metabolism in *Citrobacter*, GDH activity in wild-type and ∆*dhaD* cells was measured in the different cultures (Table 2). The activity of GDH in the mutant was negligible compared to that in the wild-type strain, confirming the *dhaD* knock-out. For the wild-type strain, the highest activity of GDH was obtained under anaerobic conditions with glycerol as sole carbon source, similar to Neijssel *et al.*[25].

**Table 2:** **Specific enzyme activity of GDH in** ***C. werkmanii*** **DSM17579 (WT) and** ***C. werkmanii*** **DSM17579 ∆** ***dhaD*** **(KO)**

	**Aerobic condition**		**Anaerobic condition**	
	**Glycerol**	**Glycerol + glucose**	**Glycerol**	**Glycerol + glucose**
WT (U/mg)	1.406 ± 0.283	0.005 ± 0.002	3.770 ± 0.259	1.293 ± 0.214
KO (U/mg)	0.007 ± 0.003	BDL	No growth	0.022 ± 0.005

Both strains were grown under (an)aerobic conditions in media with 163 mM glycerol only or 54 mM glucose and 163 mM glycerol (0.33 molar ratio glucose/glycerol) as carbon sources; BDL = below the detection limit.

Starting with 163 mM glycerol as sole carbon source, almost all glycerol is consumed or converted by *C. werkmanii* DSM17579. This strain produces only a marginal concentration of 3-HPA and has a yield of 0.6 ± 0.0 mol PDO/mol glycerol with a final PDO concentration of 97.6 ± 1.9 mM (Table 3). The remaining 65.4 mM glycerol, not converted in PDO, is converted by GDH to DHA and further channeled into the glycolysis. The GDH enzyme activity for this strain was of 3.8 ± 0.3 U/mg protein under these conditions. When *C. werkmanii* DSM17579 was grown on glycerol and glucose under anaerobic conditions, the GDH activity dropped to 1.3 ± 0.2 U/mg protein. However, since the enzyme is still active, this means that the wild-type not only consumes glucose, but also glycerol for growth and cell maintenance, which is confirmed by the metabolites. From the 132.5 ± 0.2 mM of glycerol consumed, 1.2 ± 0.1 mM is converted to 3-HPA and 94.9 ± 2.9 mM to PDO, leaving 27.5% of the glycerol unconsumed by GDHt, thus most likely converted by the oxidative branch of the glycerol metabolism. These observations also indicate that, under anaerobic conditions, glycerol and glucose can be taken up and metabolized simultaneously.

**Table 3:** Consumed glucose concentration, consumed glycerol concentration, PDO concentration produced, and yield on glycerol of PDO

	**Aerobic condition**		**Anaerobic condition**	
	**Glycerol**	**Glycerol + glucose**	**Glycerol**	**Glycerol + glucose**
Glucose consumed (mM)	NA	55.2 ± 0.3	NA	45.0 ± 0.1
Glycerol consumed (mM)	136.9 ± 2.7	5.9 ± 0.3	54.7 ± 3.5	132.5 ± 0.2
PDO produced (mM)	14.0 ± 0.5	2.4 ± 0.1	97.6 ± 1.9	94.9 ± 2.9
Yield (mol PDO/mol glycerol)	0.1 ± 0.0	0.4 ± 0.0	0.6 ± 0.0	0.7 ± 0.0

*C. werkmanii* DSM17579 was grown under (an)aerobic conditions in media with 163 mM glycerol only or 54 mM glucose and 163 mM glycerol (0.33 molar ratio) as carbon sources. (NA = not applicable).

Under aerobic conditions, glycerol is barely consumed by *C. werkmanii* DSM17579 (Table 3). Moreover, the activity of the GDH drops almost 3 times on glycerol and is minor on a glucose/glycerol mix, compared to the anaerobic conditions. Glucose on the other hand is completely consumed (Table 3). Hence, under aerobic conditions, glucose seems to inhibit the uptake and metabolism of glycerol. This is probably due to the regulation by the phosphoenol pyruvate:carbohydrate phosphotransferase system (PTS). A similar regulation exists in *Escherichia coli*. The central regulatory molecule in the PTS system is the soluble protein EIIA^Glc^, which can occur in two states: phosphorylated EIIA^Glc^, and non-phosphorylated EIIA^Glc^. The latter state binds and inhibits the proteins essential in the catabolism of several carbohydrates, such as lactose, melibiose, maltose, and glycerol, resulting in inhibition of uptake and subsequent consumption of these carbon sources [26]. It has been proven by Darbon *et al.*[27] for *E. coli* and *Salmonella typhimurium* that non-phosphorylated EIIA^Glc^ interacts with glycerol kinase and inhibits its activity under aerobic conditions leading to a reduced uptake of glycerol [27].

Our results and the similarities with results found for phylogenetic related micro-organisms strengthen our hypothesis that *C. werkmanii* DSM17579 has the same aerobic and anaerobic glycerol catabolic pathways as *E. coli* and *Salmonella* species. Under anaerobic conditions glycerol is consumed via GDH, which is not affected by the PTS, under aerobic conditions, glycerol is mainly consumed via glycerol kinase, which is affected by PTS and thus by the presence of glucose. Moreover, much less PDO is formed aerobically compared to under anaerobic conditions (Table 3).

### Effect of the molar ratio glucose/glycerol on the production of 1,3-propanediol

The maximum theoretical yield of PDO on glycerol increases from 0.72 to 1.0 in *K. pneumoniae* DSM2026 when the molar ratio of glucose/glycerol increases from 0.00 to 0.32 as calculated by Xiu *et al.*[19]. This implies that no hydrogen nor ethanol is formed. However, in *C. werkmanii* DSM17579 ∆*dhaD,* ethanol is still produced under anaerobic conditions on a glucose/glycerol mix with a molar ratio of 0.33 (16.39 ± 0.35 mM). Moreover, the biomass composition of our microorganism may be different compared to *K. pneumoniae*. As a consequence, the ideal molar ratio of glucose to glycerol may differ. Therefore, *C. werkmanii* DSM17579 ∆*dhaD* was grown anaerobically in cultivation medium with 33 mM glucose and several concentrations of glycerol yielding molar ratios of 1, 0.33, 0.2 and 0.1 glucose/glycerol.

**Figure 5:**
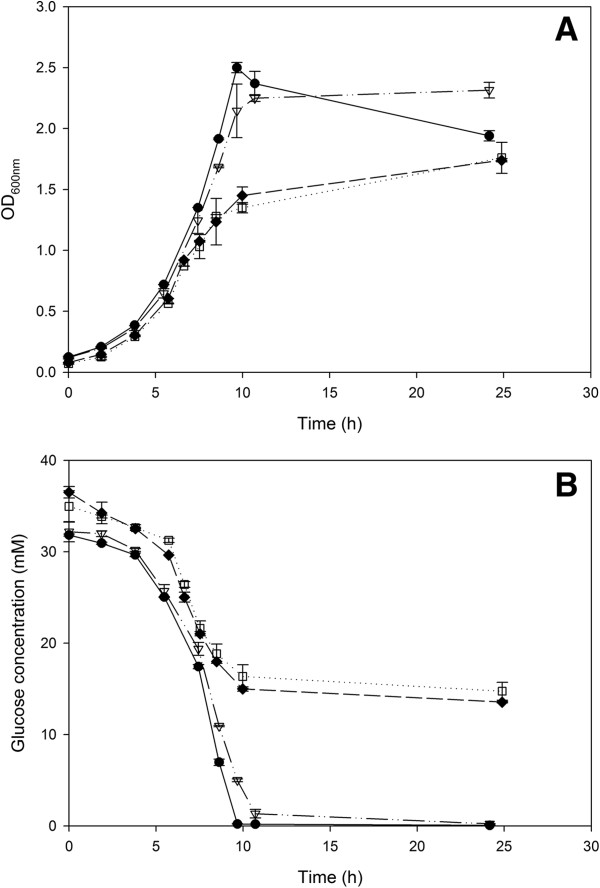
**Growth (OD**_**600nm**_**) (A) and glucose consumption (mM) (B) on different glucose/glycerol molar ratios.***C. werkmanii* DSM17579 ∆*dhaD* were grown on flask scale under anaerobic conditions in 1 (●, full line), 0.33 (∇, dash-dotted line), 0.2 (♦, dashed line), and 0.1 (□, dotted line) mol glucose/mol glycerol. The errors represent the standard deviation calculated from 2 independent experiments.

The growth (Figure 5A) and the glucose uptake (Figure 5B) in the media with the molar ratios 0.1 and 0.2 decreases after 7.5 h, and even ceases after 10 h, which is also reflected in a lower final OD_600nm_ compared to the molar ratios 0.33 and 1. In the end, still 15 mM glucose is present in the medium and the final pH does not differ for the molar ratios 0.1 and 0.2 than for the other cultures (Table 4). The yield of PDO on glycerol decreases significantly going from a molar ratio of 0.33 to 0.2 (Table 4), suggesting that the growth slowdown is due to the formation of the intermediate 3-HPA.

**Table 4:** Effect of the glucose/glycerol ratio on pH and yield; and carbon balance for glycerol utilization

**Molar ratio glucose/glycerol**	**Yield (mol PDO/mol glycerol)**	**pH**	**Carbon balance glycerol (%)**
1	0.90 ± 0.04	5.68 ± 0.05	95.51 ± 0.13
0.33	0.68 ± 0.05	6.07 ± 0.06	109.09 ± 1.26
0.2	0.43 ± 0.12	5.73 ± 0.24	99.80 ± 1.42
0.1	0.37 ± 0.07	5.94 ± 0.02	99.03 ± 0.35

*C. werkmanii* DSM17579 ∆*dhaD* was grown in medium with several molar ratios glucose/glycerol under anaerobic conditions on flask scale. The errors represent the standard deviation calculated from two independent experiments.

It is reported that in aqueous solutions, 3-HPA undergoes a reversible dimerization and hydration, resulting in a mixture of 3-hydroxypropionaldehyde (3-HPA), 1,1,3-trihydroxypropane (HPA hydrate), and 2-(2-hydroxyethyl)-4-hydroxy-1,3-dioxane (HPA dimer), called HPA, the HPA system or reuterin. This HPA system is an antimicrobial compound with activity towards a wide range of pathogens and food spoilage organisms, including both gram-positive and gram-negative bacteria, yeasts, moulds, and protozoa [28]. Depending on the total concentration of the HPA system, more HPA dimer (4.9 M HPA or higher) or HPA hydrate (0.03 M or lower) is present [29]. Since our medium is an aqueous solution, HPA is present under the three types, but to avoid confusion they will all be named 3-HPA in this article.

**Figure 6:**
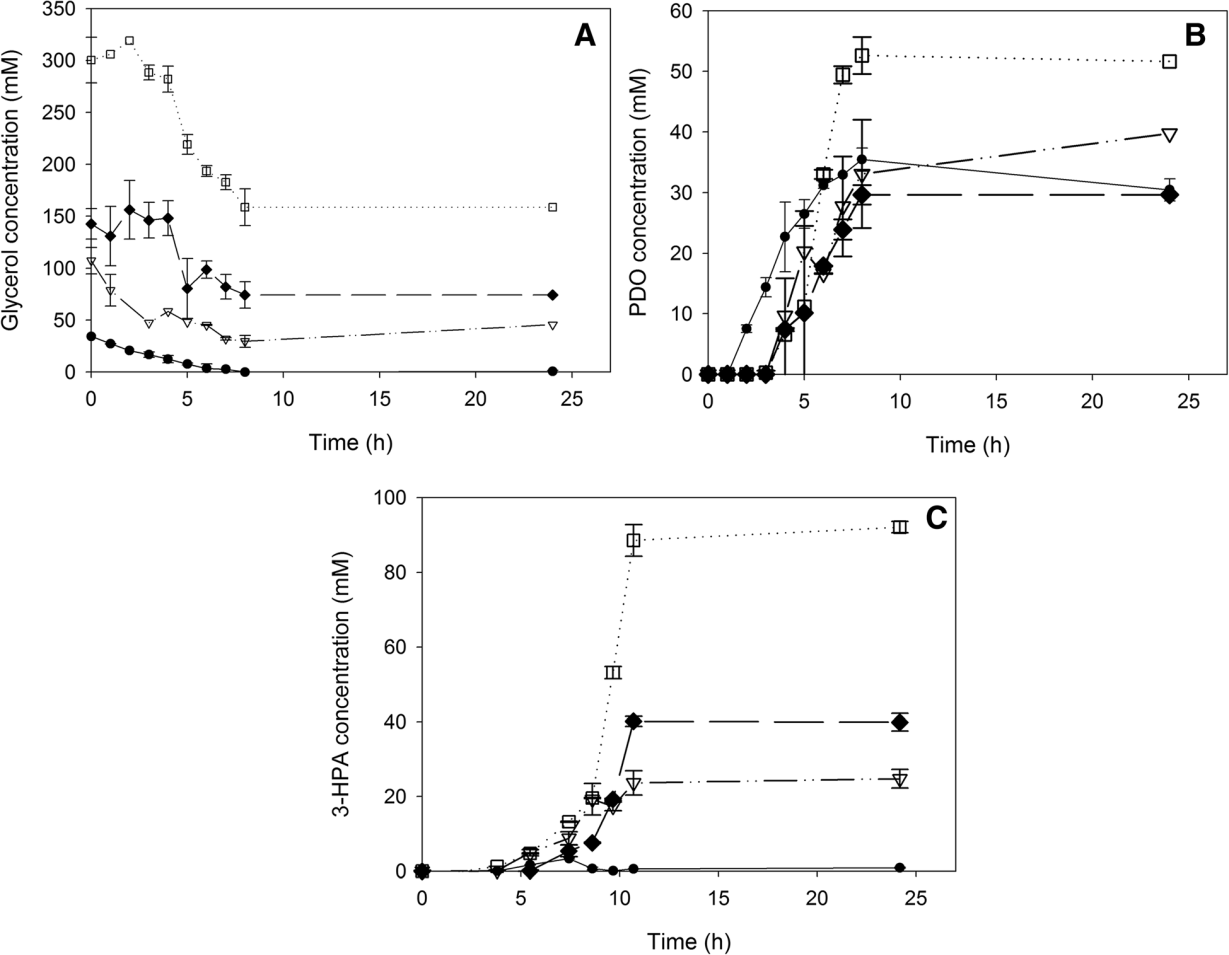
**Glycerol (A), PDO (B) and 3-HPA (C) concentration (mM) on different glucose/glycerol ratios.***C. werkmanii* DSM17579 ∆*dhaD* was grown on flask scale under anaerobic conditions in medium with 1 (●, solid line), 0.33 (∇, dash-dotted line), 0.2 (♦, dashed line), and 0.1 (□, dotted line) mol glucose/mol glycerol. The errors represent the standard deviation calculated from 2 independent experiments.

Barbirato *et al.*[30] noticed a growth and PDO production stop after consumption of about 430 mM glycerol by *Enterobacter agglomerans* CNCM1210, due to 3-HPA accumulation. They also investigated the 3-HPA accumulation in *K. pneumoniae* and *C. freundii*: both species accumulate 3-HPA until a level of, respectively, 24 mM and 17 mM is reached, after which it is further converted to PDO, allowing growth and glycerol consumption to continue until glycerol is depleted [30]. The same effect was observed for *C. werkmanii* DSM17579 during glycerol inhibition tests [12]: 3-HPA accumulates first, but then decreases and is fully converted at the end of the tests. However, in the present experiments, 3-HPA is not dissimilated completely at the end, even at the molar ratio 1 (Figure 6). This indicates a discrepancy in the cofactor balance of the PDO production. In PDO-producing microorganisms, the first step of the oxidative pathway (*i.e*. glycerol consumption for growth and maintenance), provides NADH which is used in the last step of the reductive pathway forming PDO. In the wild-type grown on glycerol and a co-substrate, glycerol will partly enter the oxidative pathway yielding more NADH as when grown on the co-substrate as sole carbon source. As such, likely enough reducing equivalents (NADH) are formed to convert 3-HPA entirely to PDO. However, in the case of the single knock-out strain, glycerol is not oxidized anymore as shown by the carbon balance for glycerol utilization in Table 4, resulting in a lower amount of reducing equivalents. Furthermore, ethanol, lactate and succinate which production competes for NADH, are still formed by the single knock-out on a glycerol/glucose medium (Additional file 1: Table S2). As a result of this competition and a decreased production of NADH due to the deletion of *dhaD*, an imbalance in the PDO pathway is created, and subsequently, 3-HPA is accumulated. In the case of the molar ratios glucose/glycerol of 0.1 and 0.2, 3-HPA accumulation exceeds the inhibitory concentration, which is somewhere between 25 mM 3-HPA and 40 mM 3-HPA in *C. werkmanii* DSM17579 ∆*dhaD*.

To overcome this obstruction, two possibilities come ahead: deletion of the genes competing for NADH and overexpression of PDODH. The first strategy has successfully been applied in *Klebsiella* sp. by several researchers [31-33], while the second has been fulfilled by overexpressing an NADPH-dependent PDO oxidoreductase in *E. coli*[34,35] and *K. pneumoniae*[36,37]. Therefore, multiple knock-out and overexpression mutants will be needed to be made in *C. werkmanii* DSM17579 ∆*dhaD*.

## Conclusions

In this work, the first knock-out strain has been created in *Citrobacter werkmanii*, *C. werkmanii* DSM17579 ∆*dhaD,* using a newly developed transformation and knock-out protocol. As such, the growth and production pathway for PDO production from glycerol were disconnected resulting in the need of a co-substrate for growth and cell maintenance. With a co-substrate/glycerol ratio of 0.33, glucose was the most promising co-substrate of 13 tested carbon sources. However, a competition between PDODH and NADH-consuming enzymes emerged resulting in the suggestion to make multiple knock-out mutants and overexpression mutants in *Citrobacter werkmanii* DSM17579 ∆*dhaD.*

## Methods

All chemicals were obtained from Sigma (Belgium), unless otherwise mentioned.

### Strains and plasmids

*Citrobacter werkmanii* DSM17579 was obtained from the German Collection of Microorganisms and Cell Cultures (DSMZ, Germany). Strains were preserved in a (1:1) glycerol (70% v/v):LB (Luria Broth: trypton 10 g/L, yeast extract 5 g/L, NaCl 5 g/L, Difco, BD, Belgium)-medium solution. Plasmids were maintained in *Escherichia coli* DH5α. Plasmids pKD3 and pKD4 were obtained from Prof. dr. J-P Hernalsteens (Vrije Universiteit Brussel, Belgium), while pKD46-Gm and pCP20-Gm (containing the gentamicin resistance gene *aac(3)-Id* of *Salmonella enterica*) were kindly provided by dr. Benoît Doublet (INRA, France) [14].

### Creation of *Citrobacter werkmanii* DSM17579 ∆*dhaD*

The ∆*dhaD* knock-out of *C. werkmanii* DSM17579 was created as described by Datsenko and Wanner [13] for *E. coli*, with some modifications for KO-creation in *Citrobacter* (see results and discussion section). A detailed protocol of the transformation and knock-out strategy is given in the Additional file 1.

The primers used to make the linear DNA and the control primers used to verify the deletion of the gene (“out”) are listed in Table 5. The knock-out colonies were analyzed by sequencing (LGC Genomics, Germany) using the primers Fw-*dhaD*-in-out and Rv-*dhaD*-in-out (Table 5) for loss of all antibiotic resistance markers.

**Table 5:** **Primers used to create and verify the knock-out strain** ***C. werkmanii*** **DSM17579 ∆** ***dhaD***

**Primer name**	**Sequence (5’ → 3’)**
Fw-*dhaD*-in-P1	AGAGAAGGTACTCAATGGTCTGCATAGCCATGACATTAGCTGCCACGCGGGTGTAGGCTGGAGCTGCTTC
Rv-*dhaD*-in-P2	AGCCACGGCCATGATCTTGCCATCAATGTCATCTTTTACGCCCATTTCCGCCATATGAATATCCTCCTTAG
Fw-*dhaD*-in-out	GGTCTGCATAGCCATGACATTAGC
Rv-*dhaD*-in-out	TAGCCACGGCCATGATCTTG
SPrimer 46TA	TTCCGTGTCGCCCTTATTCC
SPrimer46 TB	TAGTGTATGCGGCGACCGAG

### Growth and production characterization of *C. werkmanii* DSM17579 wild-type and knock-out mutant

Twelve sugars (L-arabinose, D-fructose, D-galactose, D-glucose, D-maltose, D-mannose, D-mannitol (Merck, Belgium), D-sorbitol, L-rhamnose, L-fucose, D-xylose, and D-ribose (Senn Chemicals, Switzeland) and DHA were tested as possible co-substrate for the production of PDO from glycerol. The medium used for the sugar tests is described by Maervoet *et al*. [12]. In order to find the optimal glucose/glycerol molar ratio, the same cultivation medium was used with 33 mM glucose and several glycerol concentrations. Molar ratios of 1, 0.33, 0.2, and 0.1 mol glucose/mol glycerol were tested. The cultivation conditions for these tests are described in Maervoet *et al*. [12]. Standard deviations were calculated from two independent experiments.

### Analytical methods

The biomass concentration was measured as absorbance at 600 nm. All sugars (except glucose), DHA, glycerol, PDO, lactate, acetate, succinate and ethanol were determined in a HPLC system (Varian, Belgium) with an Aminex HPX-87H Organic Acid Analysis Column (Bio-Rad Laboratories, Belgium), using a Refractive Index Detector and a dual Ultraviolet Detector with a wavelength of 210 nm and 265 nm. The column temperature was 65°C. A solution of 5 mM H_2_SO_4_ was used as mobile phase at 0.6 mL/min flow rate. Glucose was analyzed using the YSI 2700 SELECT Biochemistry Analyzer (YSI Life Sciences, Ankersmid Scientific, Belgium). 3-HPA was determined by a HPLC system with a Rezex ROA Organic Acid Analysis column (Phenomenex, Belgium) using a dual Ultraviolet Detector with a wavelength of 210 nm and 265 nm. The column temperature was 40°C. A solution of 10 mM H_2_SO_4_ was used as mobile phase at 0.5 mL/min flow rate.

### Detection of glycerol dehydrogenase activity

The medium containing 163 mM glycerol or 163 mM glycerol and glucose (3/1 molar ratio) and the anaerobic cultivation conditions used to grow the strains for enzyme assays are further described in Maervoet *et al*. [12]. The aerobic cultivation of the cultures was done on an orbital shaker at 200 rpm and 37°C.

The extraction process and enzyme assay to determine the glycerol dehydrogenase activity are described in Maervoet *et al*. [38]. Protein concentrations were measured with the BCA Protein Assay kit from Thermo Scientific (Belgium). All enzyme assays were performed in duplicate.

## Abbreviations

3-HPA: 3-hydroxypropionaldehyde, HPA dimer and HPA hydrate; DHA: Dihydroxyacetone; GDH: Glycerol dehydrogenase; PDO: 1,3-propanediol; PDODH: 1,3-propanediol dehydrogenase; PTS: Phosphoenol pyruvate:carbohydrate phosphotranferase system.

## Competing interests

The authors declare no commercial or financial conflict of interest.

## Authors’ contributions

VM designed and carried out this work, and drafted the manuscript. SDM supervised the research and helped to draft the manuscript. FA participated in experimental aspects of this work and edited the manuscript. JB, WS and MDM supervised the research and edited the manuscript. All authors read and approved the final version of the manuscript.

## Supplementary Material

Additional file 1: Table S1-S2, Figure S1 1,3-propanediol production with *Citrobacter werkmanii* DSM17579: effect of a *dhaD* knock-out Click here for file

## References

[B1] Moo-Young M Hirose T Geiger GH The Rheological Effects of Substrate-Additives on Fermentation Yields Biotechnol Bioeng 1969 9 725 731 5824424 10.1002/bit.260110416

[B2] Zhang Y Wang Y Wang Z-G Wang X Guo H-S Meng D-F Wong P-k Optimization of Fermentation Medium for the Production of Atrazine Degrading Strain *Acinetobacter* sp. DNS by Statistical Analysis System J Biomed Biotechnol 2012 doi:10.1155/2012/623062 10.1155/2012/623062 PMC3470899 23093851

[B3] Manivasagan P Sivasankar P Venkatesan J Senthilkumar K Sivakumar K Kim S-K Production and characterization of an extracellular polysaccharide from *Streptomyces violaceus* MM72 Int J Biol Macromol 2013 59 29 38 23597709 10.1016/j.ijbiomac.2013.04.012

[B4] Parekh S Vinci VA Strobel RJ Improvement of microbial strains and fermentation processes Appl Microbiol Biotechnol 2000 54 287 301 10.1007/s002530000403 11030563

[B5] Lin H Bennett GN San K-Y Metabolic engineering of aerobic succinate production systems in *Escherichia coli* to improve process productivity and achieve the maximum theoretical succinate yield Metab Eng 2005 7 116 127 10.1016/j.ymben.2004.10.003 15781420

[B6] Sanchez AM Bennett GN San KY Novel pathway engineering design of the anaerobic central metabolic pathway in Escherichia coli to increase succinate yield and productivity Metab Eng 2005 7 229 239 10.1016/j.ymben.2005.03.001 15885621

[B7] Jantama K Zhang X Moore JC Shanmugam KT Svoronos SA Ingram LO Eliminating Side Products and Increasing Succinate Yields in Engineered Strains of *Escherichia coli* C Biotechnol Bioeng 2008 101 881 893 10.1002/bit.22005 18781696

[B8] Maervoet VET De Mey M Beauprez J De Maeseneire S Soetaert WK Enhancing the Microbial Conversion of Glycerol to 1,3-Propanediol Using Metabolic Engineering Org Process Res Dev 2011 15 189 202 10.1021/op1001929

[B9] Pirie CM De Mey M Prather KLJ Ajikumar PK Integrating the Protein and Metabolic Engineering Toolkits for Next-Generation Chemical Biosynthesis ACS Chem Biol 2013 8 662 672 10.1021/cb300634b 23373985

[B10] Dodge TC Valle F **Uncoupled productive and catabolic host cell pathways** Book Uncoupled productive and catabolic host cell pathways 2002 vol. WO/2002/081631 Palo Alto: Genencor international, Inc

[B11] Liu H Ou X Zhou S Liu D Chen GQ Microbial 1,3-Propanediol, Its Copolymerization with Terephthalate, and Applications Plastics from Bacteria: Natural Functions and Applications 2010 Bejing: Springer Steinbüchel A (Series Editor): *Microbiology Monographs*

[B12] Maervoet V Beauprez J De Maeseneire S Soetaert W De Mey M *Citrobacter werkmanii*, a new candidate for the production of 1,3-propanediol: strain selection and carbon source optimization Green Chem 2012 14 2168 2179 10.1039/c2gc35369e

[B13] Datsenko KA Wanner BL One-step inactivation of chromosomal genes in *Escherichia coli* K12 using PCR products Proc Natl Acad Sci U S A 2000 97 6640 6645 10.1073/pnas.120163297 10829079 PMC18686

[B14] Doublet B Douard G Targant H Meunier D Madec JY Cloeckaert A Antibiotic marker modifications of lambda Red and FLP helper plasmids, pKD46 and pCP20, for inactivation of chromosomal genes using PCR products in multidrug-resistant strains J Microbiol Methods 2008 75 359 361 10.1016/j.mimet.2008.06.010 18619499

[B15] Derbise A Lesic B Dacheux D Ghigo JM Carniel E A rapid and simple method for inactivating chromosomal genes in *Yersinia* FEMS Immunol Med Microbiol 2003 38 113 116 10.1016/S0928-8244(03)00181-0 13129645

[B16] Jin P Li SA Lu SG Zhu JG Huang H Improved 1,3-propanediol production with hemicellulosic hydrolysates (corn straw) as cosubstrate: Impact of degradation products on *Klebsiella pneumoniae* growth and 1,3-propanediol fermentation Bioresour Technol 2011 102 1815 1821 10.1016/j.biortech.2010.09.048 21036601

[B17] Tong IT Liao HH Cameron DC 1,3-propanediol production by *Escherichia coli* expressing genes from the *Klebsiella pneumoniae* DHA regulon Appl Environ Microbiol 1991 57 3541 3546 1785929 10.1128/aem.57.12.3541-3546.1991 PMC184009

[B18] Matsuoka Y Shimizu K Catabolite regulation analysis of Escherichia coli for acetate overflow mechanism and co-consumption of multiple sugars based on systems biology approach using computer simulation J Biotechnol 2013 168 155 173 10.1016/j.jbiotec.2013.06.023 23850830

[B19] Xiu ZL Chen X Sun YQ Zhang DJ Stoichiometric analysis and experimental investigation of glycerol-glucose co-fermentation in *Klebsiella pneumoniae* under microaerobic conditions Biochem Eng J 2007 33 42 52 10.1016/j.bej.2006.09.027

[B20] Dacunha MV Foster MA Sugar-glycerol cofermentations in *Lactobacilli:* The fate of lactate J Bacteriol 1992 174 1013 1019 1732191 10.1128/jb.174.3.1013-1019.1992 PMC206182

[B21] Jayes W The Sugar engineers 2014 [http://www.sugartech.co.za/sugarprice/]

[B22] Gould BW Understanding dairy markets: Your source for market information and price risk management principles 2014 [http://future.aae.wisc.edu]

[B23] Horng YT Chang KC Chou TC Yu CJ Chien CC Wei YH Soo PC Inactivation of *dhaD* and *dhaK* abolishes by-product accumulation during 1,3-propanediol production in *Klebsiella pneumoniae* J Ind Microbiol Biotechnol 2010 37 707 716 10.1007/s10295-010-0714-9 20379761

[B24] Seo MY Seo JW Heo SY Baek JO Rairakhwada D Oh BR Seo PS Choi MH Kim CH Elimination of by-product formation during production of 1,3-propanediol in *Klebsiella pneumoniae* by inactivation of glycerol oxidative pathway Appl Microbiol Biotechnol 2009 84 527 534 10.1007/s00253-009-1980-1 19352645

[B25] Neijssel OM Hueting S Crabbendam KJ Tempest DW Dual pathways of glycerol assimilation in *Klebsiella aerogenes* NCIB 418: Their regulation and possible functional significance Arch Microbiol 1975 104 83 87 10.1007/BF00447304 1156097

[B26] Deutscher J Francke C Postma PW How phosphotransferase system-related protein phosphorylation regulates carbohydrate metabolism in bacteria Microbiol Mol Biol Rev 2006 70 939 1031 10.1128/MMBR.00024-06 17158705 PMC1698508

[B27] Darbon E Ito K Huang HS Yoshimoto T Poncet S Deutscher J Glycerol transport and phosphoenolpyruvate-dependent enzyme I- and HPr-catalysed phosphorylation of glycerol kinase in *Thermus flavus* Microbiol-Uk 1999 145 3205 3212 10.1099/00221287-145-11-3205 10589729

[B28] Doleyres Y Beck P Vollenweider S Lacroix C Production of 3-hydroxypropionaldehyde using a two-step process with *Lactobacillus reuteri* Appl Microbiol Biotechnol 2005 68 467 474 10.1007/s00253-005-1895-4 15682289

[B29] Vollenweider S Grassi G Konig I Puhan Z Purification and structural characterization of 3-hydroxypropionaldehyde and its derivatives J Agric Food Chem 2003 51 3287 3293 10.1021/jf021086d 12744656

[B30] Barbirato F Grivet JP Soucaille P Bories A 3-Hydroxypropionaldehyde, an inhibitory metabolite of glycerol fermentation to 1,3-propanediol by enterobacterial species Appl Environ Microbiol 1996 62 1448 1451 8919810 10.1128/aem.62.4.1448-1451.1996 PMC167915

[B31] Xu YZ Guo NN Zheng ZM Ou XJ Liu HJ Liu DH Metabolism in 1,3-Propanediol Fed-Batch Fermentation by a D-Lactate Deficient Mutant of *Klebsiella pneumoniae* Biotechnol Bioeng 2009 104 965 972 10.1002/bit.22455 19572314

[B32] Yang G Tian JS Li JL Fermentation of 1,3-propanediol by a lactate deficient mutant of *Klebsiella oxytoca* under microaerobic conditions Appl Microbiol Biotechnol 2007 73 1017 1024 16960737 10.1007/s00253-006-0563-7

[B33] Zhang XM Li Y Zhuge B Tang XM Shen W Rao ZM Fang HY Zhuge J Construction of a novel recombinant *Escherichia coli* strain capable of producing 1,3-propanediol and optimization of fermentation parameters by statistical design World J Microbiol Biotechnol 2006 22 945 952 10.1007/s11274-006-9139-z

[B34] Tang XM Tan YS Zhu H Zhao K Shen W Microbial Conversion of Glycerol to 1,3-Propanediol by an Engineered Strain of *Escherichia coli* Appl Environ Microbiol 2009 75 1628 1634 10.1128/AEM.02376-08 19139229 PMC2655474

[B35] Emptage M Haynie SL Laffend L Pucci JP Whited G **Process for the biological production of 1,3-propanediol with high titer** Book Process for the biological production of 1,3-propanediol with high titer 2003 US 6,514,733 B1 US: E.I. DuPont de Nemours and Company

[B36] Chen Z Liu HJ Liu DH Metabolic pathway analysis of 1,3-propanediol production with a genetically modified *Klebsiella pneumoniae* by overexpressing an endogenous NADPH-dependent alcohol dehydrogenase Biochem Eng J 2011 54 151 157 10.1016/j.bej.2011.02.005

[B37] Zhuge B Zhang C Fang HY Zhuge JA Permaul K Expression of 1,3-propanediol oxidoreductase and its isoenzyme in *Klebsiella pneumoniae* for bioconversion of glycerol into 1,3-propanediol Appl Microbiol Biotechnol 2011 87 2177 2184 20499228 10.1007/s00253-010-2678-0

[B38] Maervoet VET De Maeseneire SL Soetaert WK De Mey M Unraveling the *dha* cluster in *Citrobacter werkmanii*: comparative genomic analysis of bacterial 1,3-propanediol biosynthesis clusters Bioprocess Biosystems Eng 2014 37 711 718 10.1007/s00449-013-1041-0 23996279

