# Supplementary material for: 1,3-propanediol production with Citrobacter werkmanii DSM17579: effect of a dhaD knock-out
Source: Microb Cell Fact. 2014 May 17;13:70. doi: 10.1186/1475-2859-13-70 (PMC4031495; doi:10.1186/1475-2859-13-70)
Supplement: Additional file 1: Table S1-S2, Figure S1 — 1,3-propanediol production with Citrobacter werkmanii DSM17579: effect of a dhaD knock-out [file 1475-2859-13-70-S1.pdf]

1,3-propanediol production with *Citrobacter werkmanii* DSM17579: effect of a *dhaD* knock-out

Veerle ET Maervoet[1, *], Sofie L De Maeseneire, Fatma G Avci[2], Joeri Beauprez, Wim K Soetaert and Marjan De Mey

Centre of Expertise - Industrial Biotechnology and Biocatalysis, Department of Biochemical and Microbial Technology, Ghent University, Coupure links 653, B-9000 Ghent, Belgium; veerle.maervoet@ugent.be; sofie.demaeseneire@ugent.be; gizemavci@gmail.com; joeri.beauprez@ugent.be; wim.soetaert@ugent.be; marjan.demey@ugent.be

[1]*Present address: Laboratory of Biochemistry and Brewing, Department of Applied Bioscience Engineering, Ghent University, Valentin Vaerwyckweg 1, 9000 Ghent*

[2]*Present address: Bioengineering Department, Faculty of Engineering, Ege University, 35100 Bornova-Izmir, Turkey*

[*]*Corresponding author:*

Veerle Maervoet

Phone: +32 92 64 60 31

Fax: +32 92 42 42 79

e-mail: Veerle.Maervoet@UGent.be

Table S1. Antibiotic profile of *C. werkmanii* DSM17579. The strain was grown on different concentrations of ampicillin, kanamycin, chloramphenicol and gentamicin. +: growth; -: no growth of the bacterium after 24 h of incubation at 37 °C.

**Antibioticum**	**Concentration (µg/mL)**	**Growth**
Ampicillin	100	+
	150	+
	200	+
Kanamycin	50	-
Chloramphenicol	25	-
Gentamicin	25	-
	50	-

Figure S1. Comparison of acetate (A), ethanol (B), lactate (C) and succinate (D) concentration (M). *C. werkmanii* DSM17579 Δ*dhaD* was grown in minimal medium with different substrates (0.065 M, black) or with glycerol (0.163 M) and different co-substrates (0.33 molar ratio co-substrate/glycerol, white) on flask scale under anaerobic conditions. The errors represent the standard deviation calculated from 2 independent experiments.

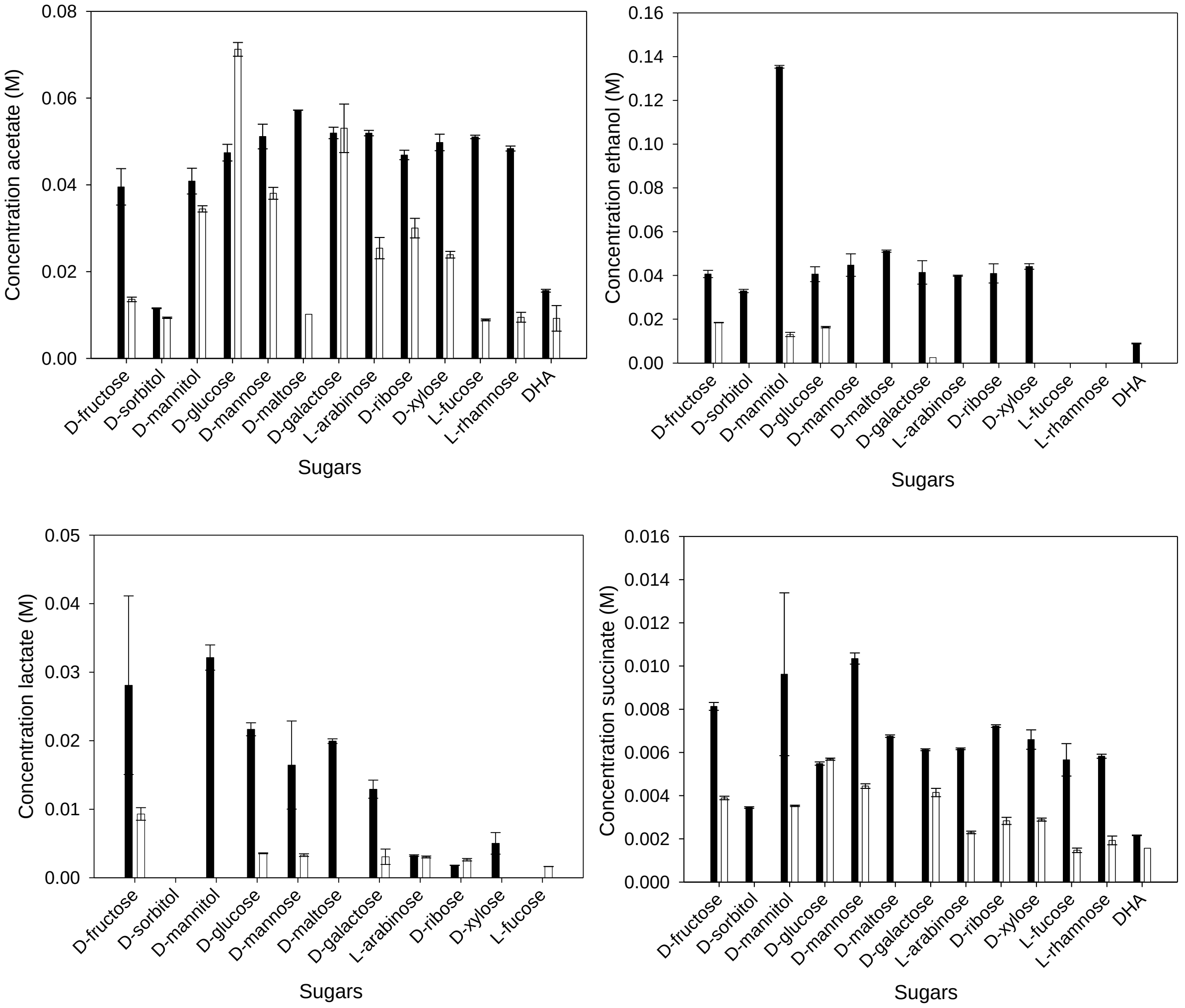

Table S2. Byproduct formation of *C. werkmanii* DSM17579 Δ*dhaD* on different glucose/glycerol molar ratios. The knock-out strain was grown on flask scale under anaerobic conditions. The errors represent the standard deviation calculated from 2 independent experiments.

Glucose/glycerol ratio	Acetate (mM)	Ethanol (mM)	Lactate (mM)	Succinate (mM)
1	40.8 ± 0.1	19.7 ± 0.6	4.4 ± 0.1	3.3 ± 0.0
0.33	46.2 ± 0.6	18.0 ± 1.2	3.1 ± 0.5	4.4 ± 0.3
0.2	37.9 ± 3.2	15.8 ± 1.4	0	4.8 ± 0.3
0.1	22.2 ± 1.0	12.6 ± 0.1	0	4.8 ± 0.1

Detailed, optimized protocol for the creation of a *dhaD* knock-out in *Citrobacter werkmanii* DSM17579

Linear double-stranded DNA The linear dsDNA amplicons were obtained by PCR using pKD3 (containing the chloramphenicol resistance cassette) and pKD4 (including the kanamycin resistance cassette) as templates and the high-fidelity PCR Master (Roche, Belgium) as master mix. Twenty nucleotides of the primers Fw-*dhaD*-in-P1 and Rv-*dhaD*-in-P2 (Table 5) were complementary to the template and flanked with 50 nt complementary to the gene to be deleted. The PCR products were PCR-purified (QIAquick PCR purification kit, Qiagen, Netherlands), digested with *Dpn*I (New England Biolabs, Bioké, Belgium), repurified from an agarose gel (QIAquick Gel extraction kit, Qiagen, Netherlands), and suspended in elution buffer (10 mM Tris-Cl, pH 8.5).

Making of electrocompetent cells and electroporation of the cells Cells grown overnight in 5 mL LB with the appropriate antibiotic were transferred to 25 mL LB with the appropriate antibiotic to have an initial OD_{600nm} of 0.05. After incubating this culture to OD_{600nm} 0.6, 10 mL of the cells were rested on ice for 30 min to stop the metabolism. Thereafter, the cells were washed with 45 mL ice-cold Milli-Q (MQ)-water, a first time, and 1 mL ice-cold MQ-water, a second time, to remove the salts. After resuspending the cells in 50 µL ice-cold MQ-water, plasmid (100 ng) or linear dsDNA (400 ng) was added. The mixture was then transferred to an electroporation cuvette and electroporation was done using a Gene Pulser TM (BioRad, Belgium) (200 Ω, 25 µFD, and 250 V). Thereafter, 1 mL LB was added to the cells, after which they were incubated for 1 h when plasmid DNA was electroporated or 3 h when linear DNA was applied, and spread onto LB-agar containing the appropriate antibioticum. The plates were incubated at the appropriate temperature until colonies were formed (typically 16 h).

Preparation of the strain to insert linear dsDNA The cells were grown without antibiotics at 37 °C to an OD_{600nm} of 0.6. After making the cells electrocompetent, they were transformed with pKD46-Gm. Thereafter, they were incubated for 1 h on an orbital shaker at 200 rpm at 30 °C, and spread onto LB-agar containing 50 µg/mL gentamicin, respectively. The gentamicin-resistant colonies were selected and tested for the presence of pKD46-Gm by PCR with the primers SPrimer46TA and SPrimer46TB (Table 5).

Transformation of the strain with linear dsDNA The cells with pKD46-Gm were grown with gentamicin (50 µg/mL) and 20 µM L-arabinose on an orbital shaker at 200 rpm at 30 °C to an OD_{600nm} of 0.6. After making the cells electrocompetent, they were transformed with linear dsDNA. Thereafter, the cells were incubated on an orbital shaker at 200 rpm at 37 °C for 3 h,

and spread onto LB-agar containing 25 µg/mL chloramphenicol (when the linear DNA was amplified from pKD3) or 50 µg/mL kanamycin (when it was amplified from pKD4). The resistant colonies were verified by PCR with control primers on the homology regions (Fw-*dhaD*-in-out and Rv-*dhaD*-in-out, Table 5).

Deletion of the antibiotic marker The selected mutants (chloramphenicol- or kanamycin-resistant) were grown on an orbital shaker at 200 rpm at 37 °C to an OD_{600nm} of 0.6. Then, they were made electrocompetent and transformed with pCP20-Gm. The cells were again grown for 1 h on an orbital shaker at 200 rpm at 30 °C, and spread on LB-agar containing 50 µg/mL gentamicin. The resistant colonies were selected and tested by PCR using the control primers and by sequencing (LGC Genomics, Germany).
